# Supplementary material for: Evidence of nematic order and nodal superconducting gap along [110] direction in RbFe2As2
Source: Nat Commun. 2019 Mar 4;10:1039. doi: 10.1038/s41467-019-08962-z (PMC6399313; doi:10.1038/s41467-019-08962-z)
Supplement: Supplementary file 1 — Supplementary Information [file 41467_2019_8962_MOESM1_ESM.pdf]

Supplementary Information for

Evidence of nematic order and nodal superconducting gap along [110] direction in $RbFe_2As_2$

X. Liu, R. Tao, M. Ren *et al*

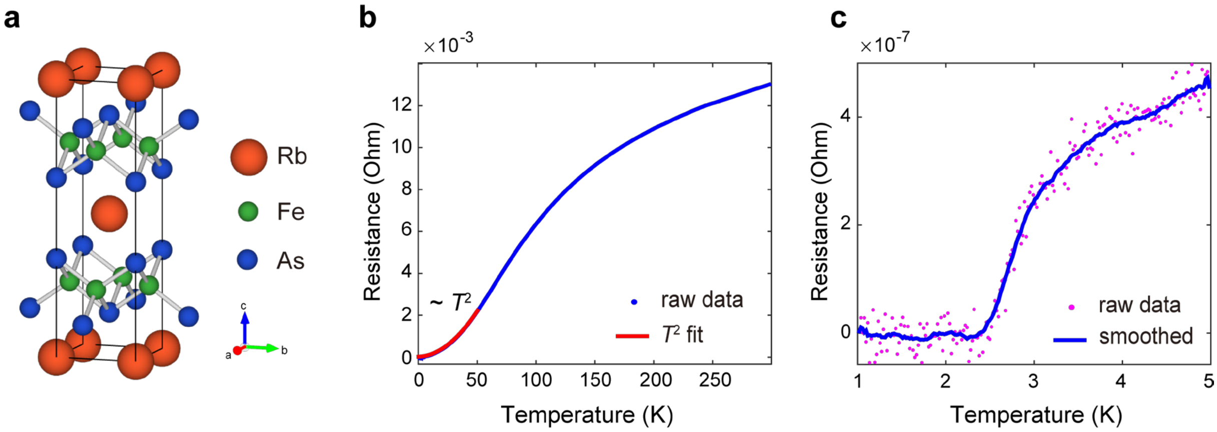

Supplementary Figure 1 | Crystal structure and transport measurement of $RbFe_2As_2$ (**a**) Bulk crystal structure of $RbFe_2As_2$. (**b**) Temperature dependence of the resistance (0 ~ 300K), the red curve below T = 50 K is a fit to T^2. (**c**) Temperature dependence of the resistance across the superconducting transition at T_c ~2.5 K.

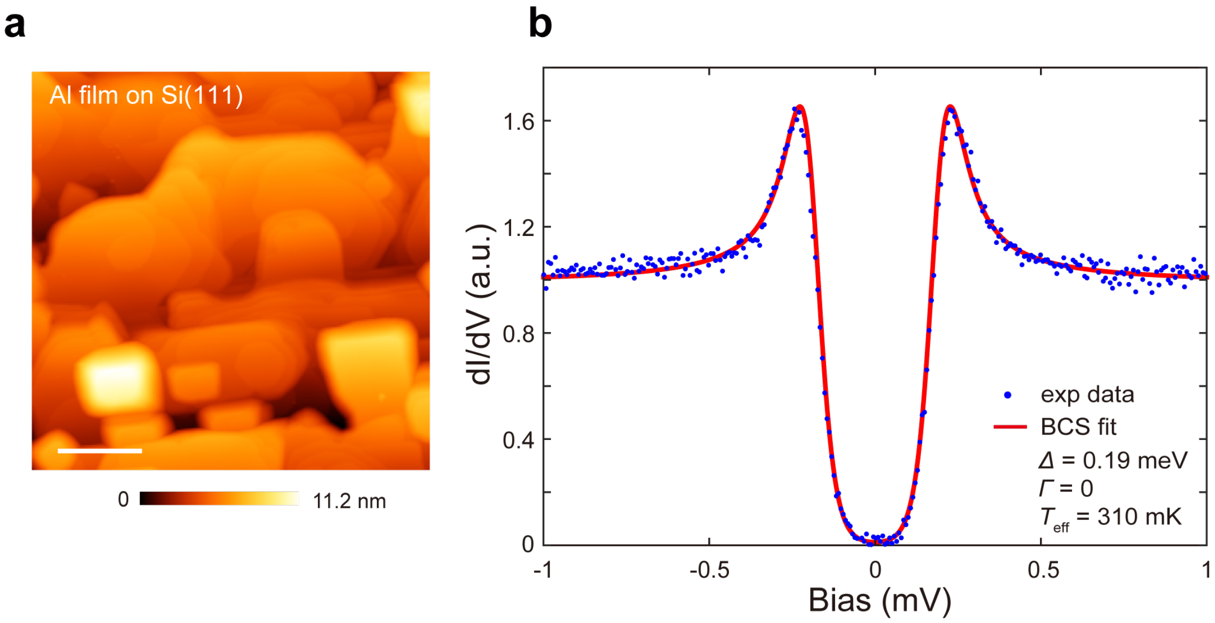

Supplementary Figure 2 | Calibration of the T_{eff} (**a**) Topographic image of an Al/Si(111) film of thickness ~20 ML (scale bar: 50 nm). (**b**) The superconducting gap of the Al/Si(111) film taken at T = 20 mK (V_b = 1 mV, I = 100 pA, ΔV = 30 μV). Red curve is the BCS fit with Δ = 0.19 meV, T_{eff} = 310 mK and Γ = 0.

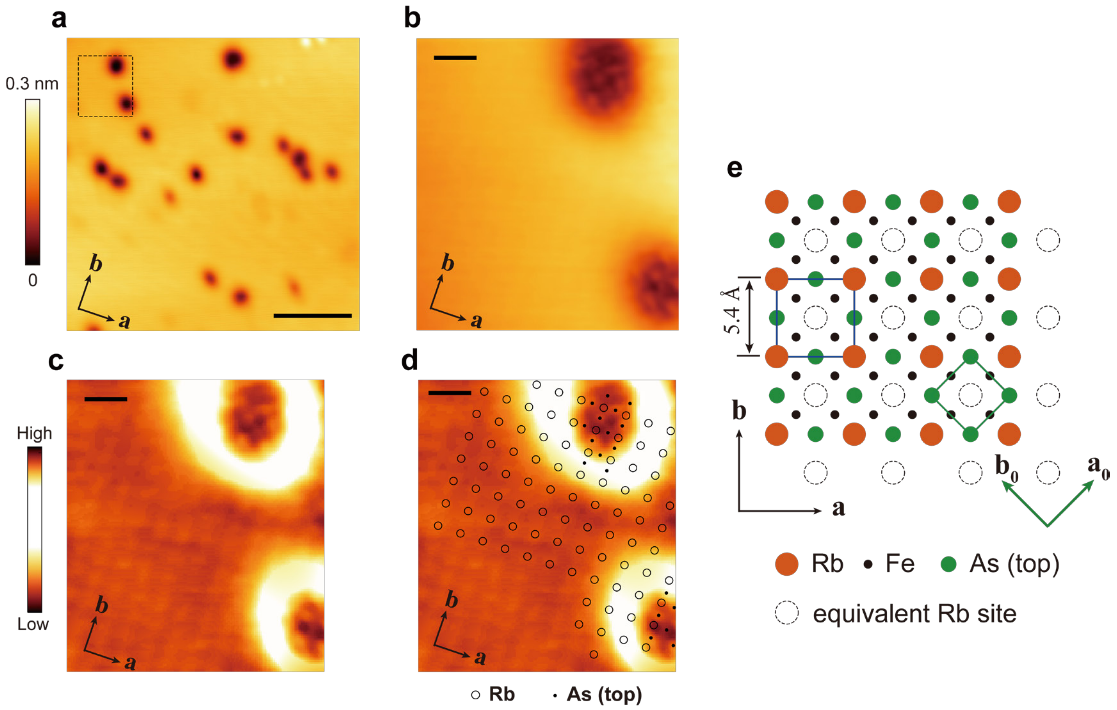

Supplementary Figure 3 | Surface atomic lattice of $RbFe_2As_2$ (**a**) An STM image of type B surface (V_b = 0.8V, I = 100pA, scale bar: 10 nm) B. (**b**) Higher-resolution image (V_b = 6mV, I = 3nA) of the region marked in panel **a**, shown with a linear color scale (scale bar: 10 nm). (**c**) The same image as panel **b** but mapped with a non-linear color scale to reveal the Rb lattice. (**d**) The same image as panel **c**, with the atomic sites marked. (**e**) The surface lattice model derived from panel **d**. The orientation of 2Fe unit cell is denoted by $\mathbf{a_0}$, $\mathbf{b_0}$.

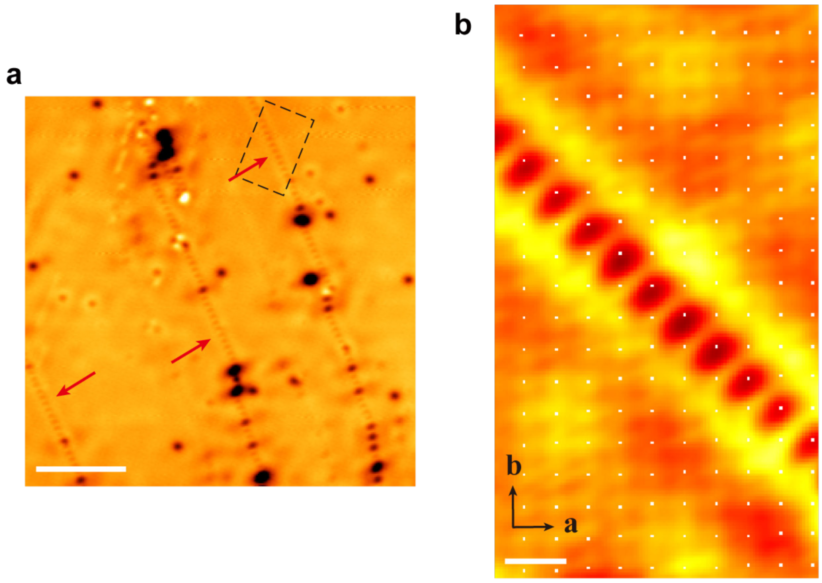

Supplementary Figure 4 | Domain structures on the surface of low temperature cleaved $RbFe_2As_2$ (a) STM image of $RbFe_2As_2$ surface cleaved at $T \approx 30$ K (V_b = -4 meV, I = 100 pA, scale bar: 10 nm). Domain boundaries are indicated by red arrows. (**b**) Atomically resolved STM image (V_b = -4 meV, I = 500 pA, scale bar: 1 nm) taken in the region marked in panel **a**. A lattice of white spots is intentionally drawn to match the atomic lattice of the lower domain. However it is misaligned from the upper domain lattice by 1/2 unit cell along **a** and **b** direction. (Images are taken at T = 4.5 K)

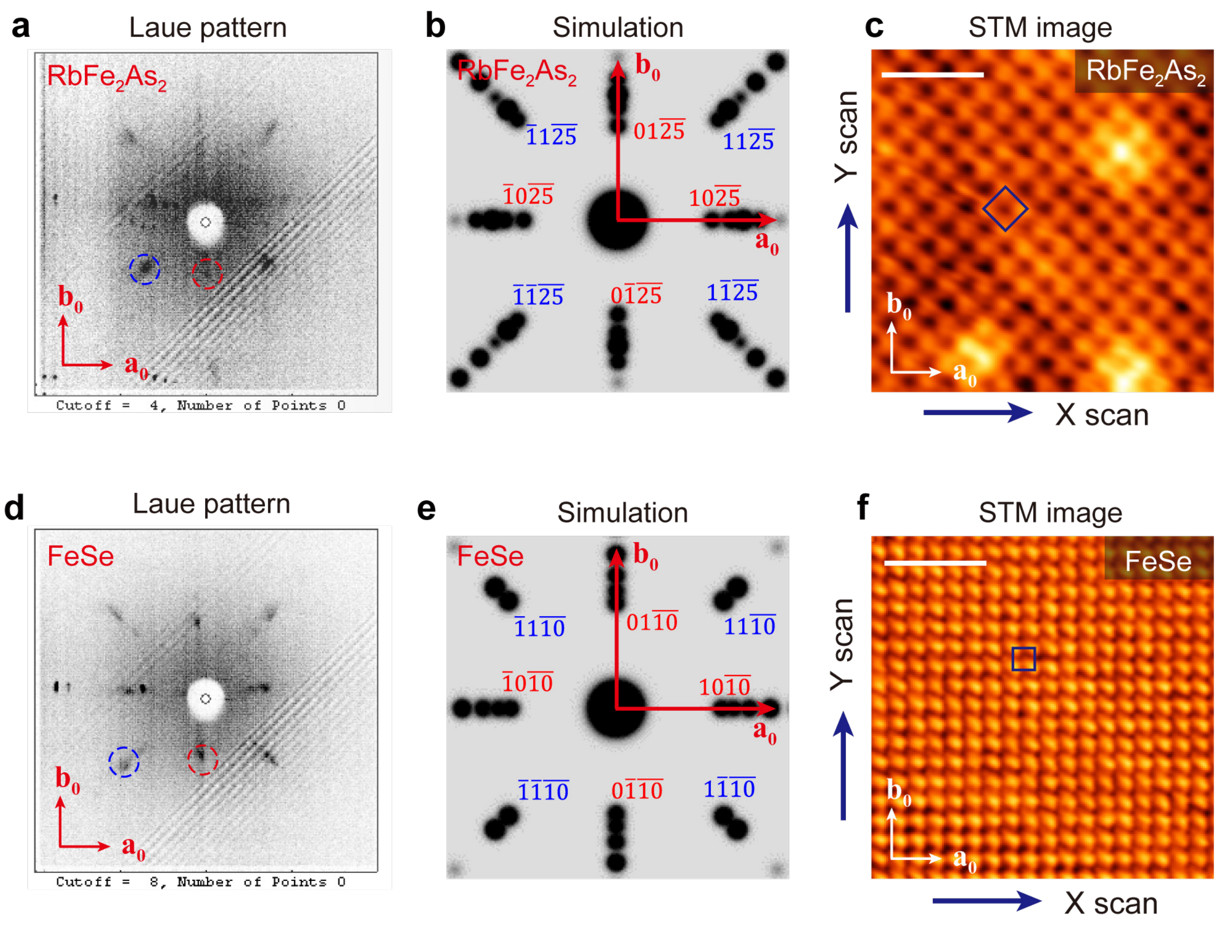

Supplementary Figure 5 | Determine the surface lattice orientation of $RbFe_2As_2$ by Laue diffraction and STM imaging. (**a**) Laue diffraction pattern of $RbFe_2As_2$ single crystal. (**b**) Simulated Laue pattern using SingleCrystal™ software for $RbFe_2As_2$ with $\mathbf{a_0}$, $\mathbf{b_0}$ along X and Y directions. The Miller indices (*hkl*) of the planes corresponding to the diffraction points near center are marked, which are belong to {01X} (red) and {11X} (blue) plane families. (**c**) STM image of cleaved $RbFe_2As_2$ with $\mathbf{a_0}$, $\mathbf{b_0}$ pre-aligned to X scan and Y scan directions (V_b = 8 mV, I = 1 nA, scale bar: 2 nm)). The lattice constant is measured to be 5.4 Å, and the lattice is rotated 45° with respect to $\mathbf{a_0}$, $\mathbf{b_0}$. (**d**) Laue diffraction pattern of a FeSe single crystal. (**e**) Simulated Laue pattern of FeSe with $\mathbf{a_0}$, $\mathbf{b_0}$ along X and Y directions. The Miller indices (*hkl*) of the plane corresponding to the diffraction points near center are also marked. (**f**) STM image of cleaved FeSe crystal with $\mathbf{a_0}$, $\mathbf{b_0}$ pre-aligned to X scan and Y scan directions (V_b = 100 mV, I = 100 pA, scale bar: 2 nm). The lattice constant is measured to be 3.75 Å, and the lattice has the same orientation with $\mathbf{a_0}$, $\mathbf{b_0}$. Note: The spots marked by red and blue circles in panels **a**, **d** are corresponding to the spots marked by the same colored indices in the simulated pattern (in panels **b**, **e**). They originated from {10X} and {11X} plane families, respectively.

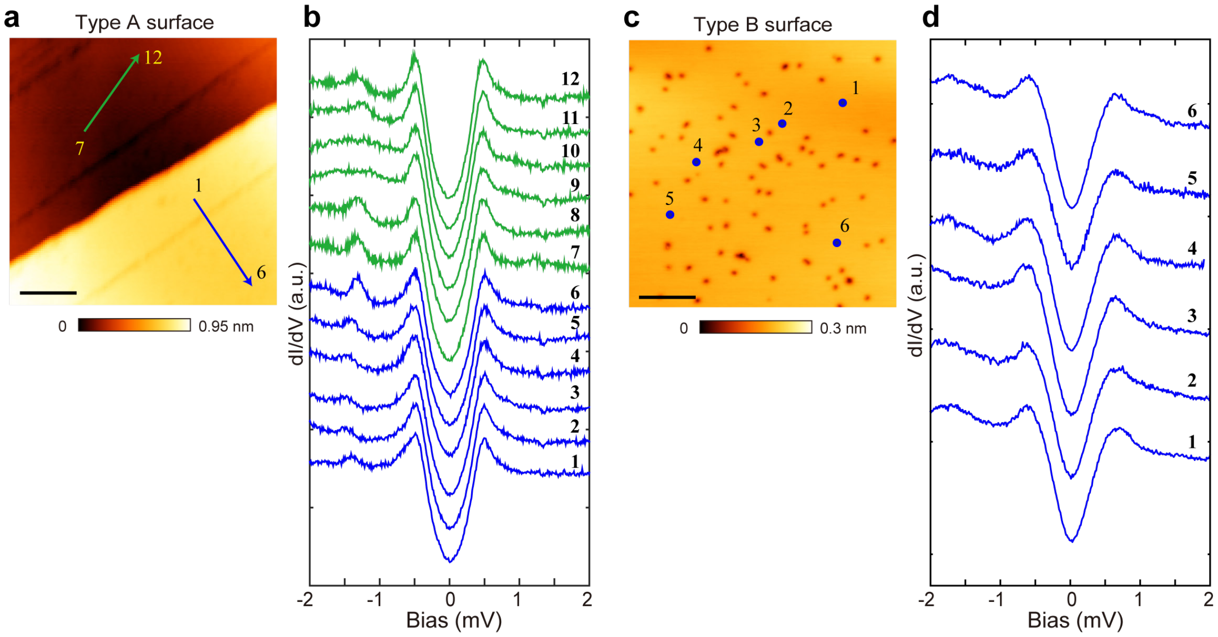

Supplementary Figure 6 | Spatial dependence of the superconducting gap on type A and B surfaces. (**a**) STM image of a type A surface (V_b = 0.5 V, I = 10 pA, scale bar: 30 nm) (**b**) superconducting gap spectra taken along the arrows marked in panel **a** (V_b = 2 mV, I = 100 pA, ΔV = 50 μV). (**c**) STM image of type B surface (V_b = 1 V, I = 10 pA, scale bar: 15 nm). (**d**) Superconducting gap spectra taken at the spots marked in panel **c** (V_b = 2 mV, I = 100 pA, ΔV = 50 μV). The spots are randomly chosen. All the spectra shown in this figure are taken at T = 20 mK and T_{eff} = 310 mK. As seen from panel **a-d**, the superconducting gaps on both type A and B surfaces are spatially homogenous.

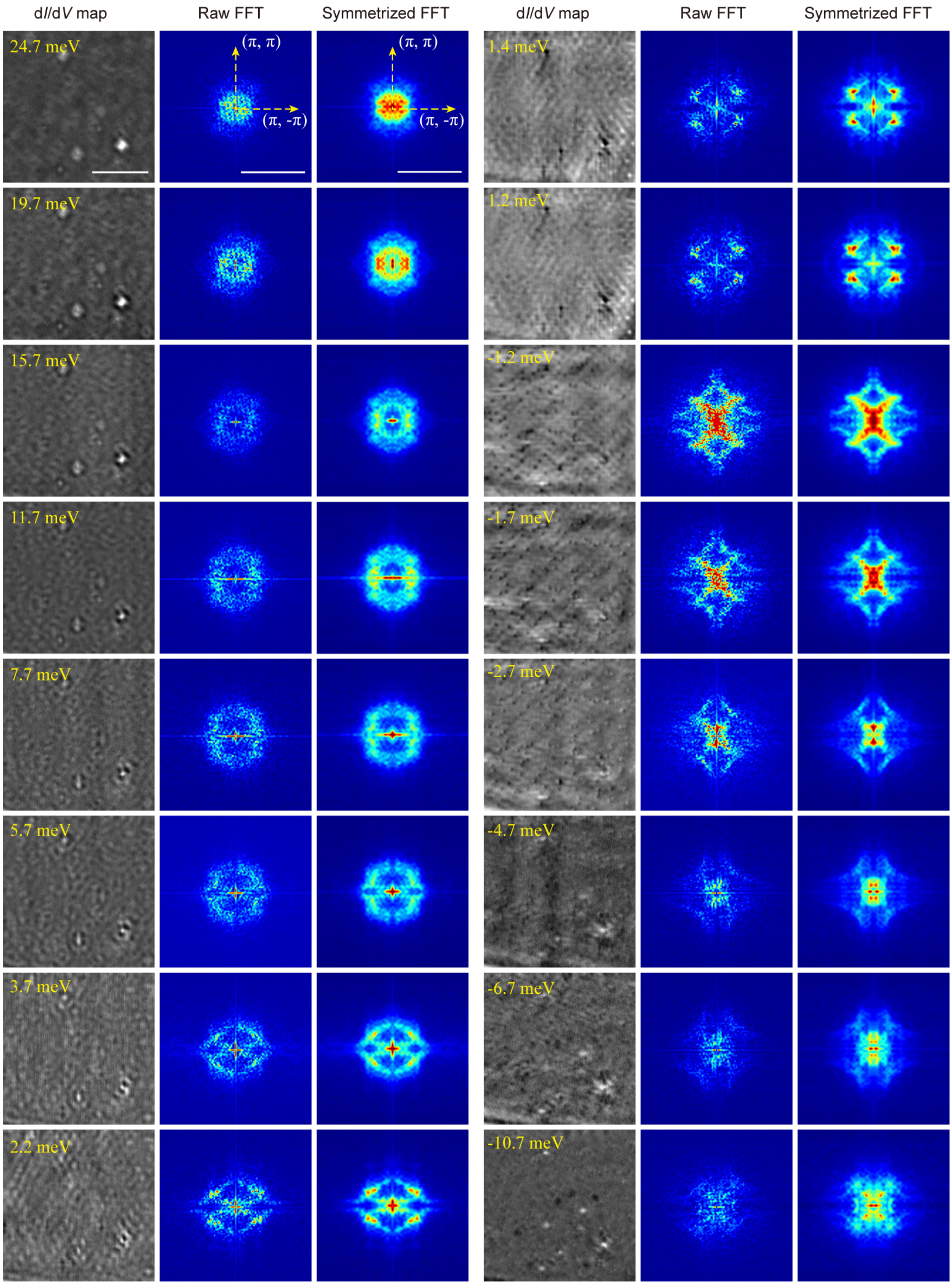

Supplementary Figure 7 | A full set of dI/dV maps taken on type A surface and their FFTs. The topography of the mapping area is shown in Fig. 1b of the main text. Each dI/dV maps are taken at a V_b equal to the mapping energy (labeled on the map) and I = 100 pA; the lock-in modulation (ΔV) for each map has an amplitude of 5% V_b. Details about the FFT symmetrization is described in Supplementary Note 3. Every dI/dV map has 256 × 256 pixels. Scale bar in the dI/dV image is 20 nm, and that in FFTs are 0.5 $Å^{-1}$.

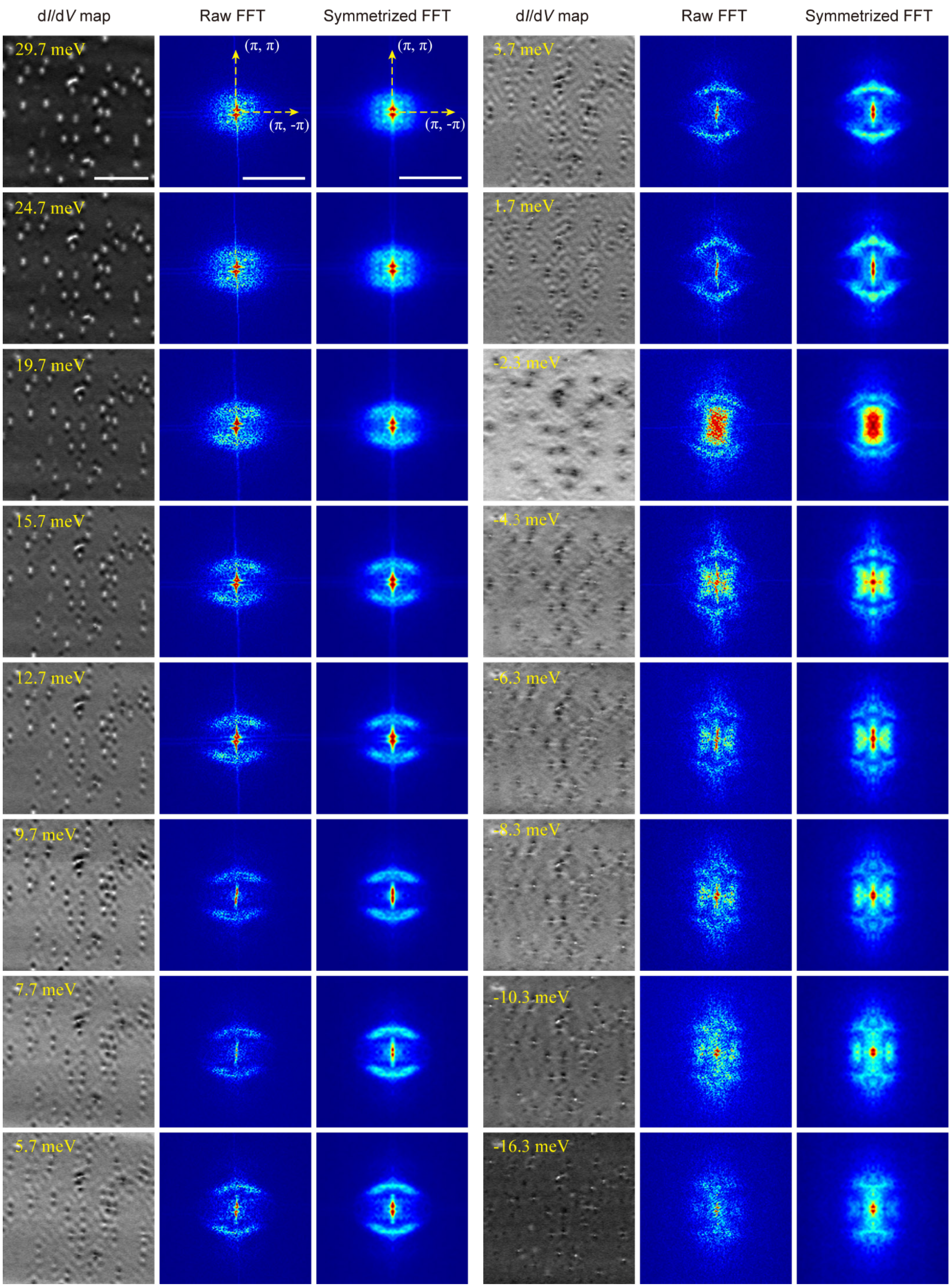

Supplementary Figure 8 | A full set of dI/dV maps taken on type B surface and their FFTs. The topography of the mapping area is shown in Fig. 1c of the main text. Each d*I*/d*V* maps are taken at a V_b equal to the mapping energy (labeled on the map) and I = 100 pA; the lock-in modulation (ΔV) for each map has an amplitude of 5% V_b. Every map has 256 × 256 pixels. Scale bar in the d*I*/d*V* image is 20 nm, and that in FFTs are 0.5 $Å^{-1}$.

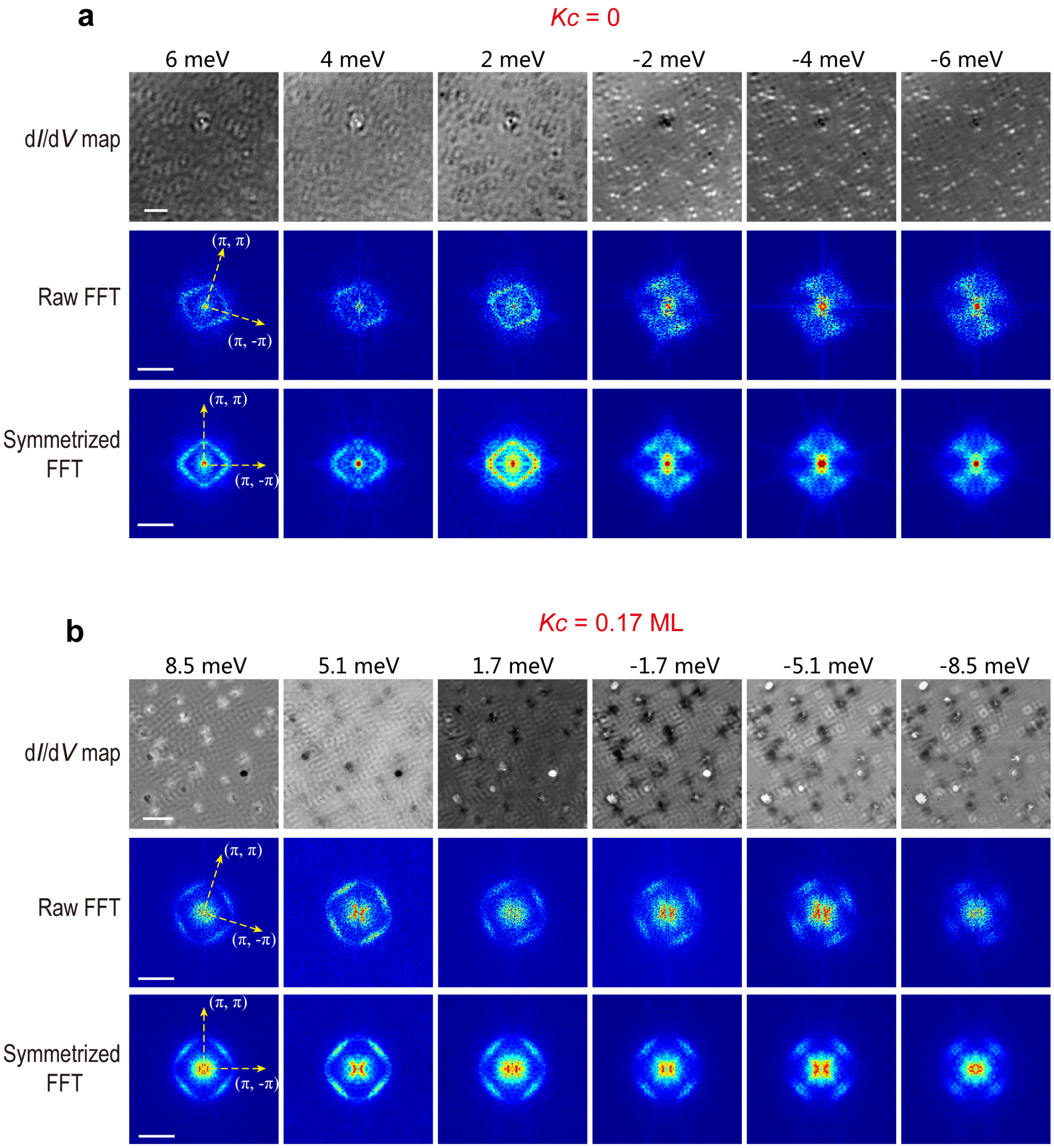

Supplementary Figure 9 | Additional QPI data of surface K-dosed $RbFe_2As_2$ (**a**) Additional d*I*/d*V* maps (scale bar: 10 nm), raw FFTs and symmetrized FFTs (scale bars: 0.3 $Å^{-1}$) taken on $RbFe_2As_2$ with *Kc* =0 at *T* = 4.5 K. All d*I*/d*V* maps are taken at the setpoint of V_b = 10 mV, *I* = 200 pA, Δ*V* = 1 mV. (**b**) Additional d*I*/d*V* maps (scale bar: 10 nm), raw FFTs and symmetrized FFTs (scale bars: 0.3 $Å^{-1}$) taken on $RbFe_2As_2$ with *Kc* = 0.17 ML at *T* = 4.5 K. All d*I*/d*V* maps are taken at the setpoint of V_b = 10 mV, *I* = 200 pA, Δ*V* = 1 mV. Each map has 250 × 250 pixels.

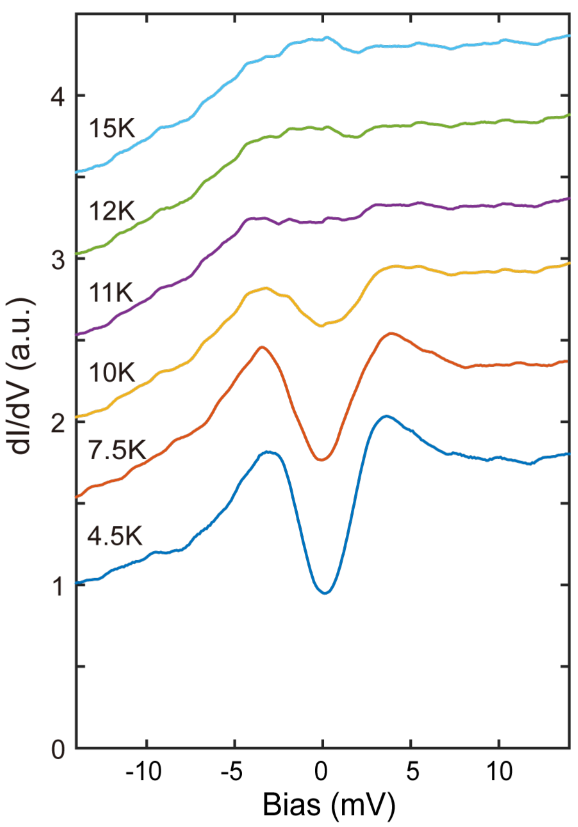

Supplementary Figure 10 | Temperature dependence of the low energy tunneling gap observed at *Kc* = 0.17 ML (V_b = 15 mV, I = 300 pA, ΔV = 1 mV for all the spectra). The gap closed at about 12 K.

Supplementary Note 1: Calibration of the effective electron temperature of the STM system

Due to the electrical noise and RF radiations, the effective electron temperature (T_{eff}) of a low-T STM is usually higher than the thermometer reading. The T_{eff} of the dilution refrigerator STM used in this work is calibrated by measuring the superconducting gap of an Al film grown Si(111). Supplementary Fig. 2a shows a typical STM image of the Al/Si(111) film, with a thickness of ~ 20 monolayer (ML), and Supplementary Fig. 2b shows its superconducting gap spectrum. A standard BCS fit (red curve) yields Δ = 0.19 meV and T_{eff} = 310 mK. Here we note that in order to make a conservative estimation of T_{eff}, Dynes broadening term is not used in the fitting (Γ = 0). In the presence of finite Γ or other broadening factors, the actual T_{eff} could be slightly lower than the fitted value.

Supplementary Note 2: Determining the surface atomic structure of cleaved $RbFe_2As_2$

The surface structure of cleaved $RbFe_2As_2$ is revealed by resolving the atomic lattice of the defect-free area and that inside of the Rb vacancies. Supplementary Fig. 3a is a typical image of type B surface with multiple Rb vacancies. Supplementary Fig. 3b shows the region marked in Supplementary Fig. 3a in greater detail (shown with a linear color scale). A square lattice inside of the Rb vacancies can be seen and has a lattice constant of ~3.8 Å, matching $\mathbf{a_0}$, which is mostly likely from the underlying FeAs layer. The lattice of the defect-free area is hard to see in Supplementary Fig. 3b due to its much smaller corrugation. To enhance the contrast, a non-linear color mapping is used in Supplementary Fig. 3c, in which both the surface Rb lattice and lattice inside the vacancy can be seen. By comparing the atomic sites of these two lattices (as marked in Supplementary Fig. 3d), the surface lattice model is derived and shown in Supplementary Fig. 3e. The surface Rb forms a √2×√2 (R45°) lattice with respect to the As lattice.

For the lattice model in Supplementary Fig. 3e, the surface Rb atoms will have another set of occupation sites, as illustrated by the dashed circles. These two equivalent occupation sites are shifted by 1/2 unit cell with respect to each other (along both **a** and **b** directions), which should result in domain structures when both are present. We indeed observed such domain structures on samples cleaved at a lower temperature (~30K), as shown in Supplementary Fig. 4a. There are domain boundaries running through the surface (marked by red arrows). Supplementary Fig. 4b is an atomically resolved image near a domain boundary. One can see aside of the boundary, the surface still displays a √2×√2 lattice. However, the lattice of the upper domain is shifted by 1/2 unit cell along **a** and **b** with respect to the lower domain. To illustrate this, we draw a lattice of white spots which matches the atomic lattice of the lower domain, however it mismatches the upper domain by the above offset. The existence of different domains gives further support to the surface lattice model in Supplementary Fig. 3e.

The assignment of surface atomic structure above is based on STM imaging. To further confirm the orientation of surface √2×√2 lattice with respect to the bulk FeAs lattice, we performed Laue diffraction measurement to accompany STM imaging. The results are summarized in Supplementary Fig. 5. We first determined the orientation of $\mathbf{a_0}$ and $\mathbf{b_0}$ (the in-plane basic vectors of 2Fe unit cell) of $RbFe_2As_2$ single crystal by comparing its measured Laue pattern with a simulated pattern. As shown in the simulation in Supplementary Fig. 5b, the four diffraction spots that closest to the center, labelled by red colored Miller indices, are originated from {10X} plane family (defined by 2Fe unit cell, so they are along the $\mathbf{a_0}$ or $\mathbf{b_0}$ directions); while the spots with blue colored Miller indices are from {11X} plane family. In the measured Laue pattern, only the spots close to the center show up with significant weight (Supplementary Fig. 5a), so the crystal orientation can be determined by comparing it to Supplementary Fig. 5b. Then the crystal was glued on STM sample holder with $\mathbf{a_0}$ and $\mathbf{b_0}$ aligned to X and Y scan directions, respectively. The STM image of cleaved surface (Supplementary Fig. 5c) then directly shows the surface √2×√2 lattice is rotated 45° with respect to $\mathbf{a_0}$ and $\mathbf{b_0}$. We also repeated the same procedure on a pure FeSe single crystal, the results are shown in Supplementary Figs. 5d-f. The FeSe has a Laue pattern in analogous to $RbFe_2As_2$, and its $\mathbf{a_0}$, $\mathbf{b_0}$ directions are determined in a similar way. The STM image in Supplementary Fig. 5f show that its surface lattice has a constant of 3.75 Å, with the direction the same as $\mathbf{a_0}$ and $\mathbf{b_0}$. This is well expected for a Se terminated surface of FeSe. Therefore, combined Laue and STM measurement directly indicates the surface √2×√2 lattice of $RbFe_2As_2$ is rotated 45° with respect to $\mathbf{a_0}$, $\mathbf{b_0}$.

Supplementary Note 3: Additional QPI data of as-cleaved and surface K-dosed $RbFe_2As_2$, and details of the FFT symmetrization process.

Additional d*I*/d*V* maps, raw FFTs and symmetrized FFTs taken on type A and B surfaces are shown in Supplementary Fig. 7 and Supplementary Fig. 8, respectively. Symmetrized FFTs are all obtained by mirror symmetrizing the raw FFTs along (π, π) and (π, -π) directions. Detailed process is 1): Identify the (π, π) and (π, -π) directions of the raw FFT by using atomically resolved images; 2): Mirror flip the raw FFT along (π, π) and add it to the raw FFT; 3) Flip the results of 2) along (π, -π) and add it to the results of 2).

Additional d*I*/d*V* maps, raw FFTs and symmetrized FFTs taken on $RbFe_2As_2$ with *Kc* =0 and *Kc* = 0.17 ML at *T* = 4.5 K are shown in Supplementary Fig. 9a-b. The symmetrized FFTs are also obtained by mirror symmetrizing the raw FFTs along (π, π) and (π, -π) directions, as described above.
